# Energy‐Efficient Fabrication of Biomimetic Materials for Sustainable Infrastructure Applications

**DOI:** 10.1002/advs.202503854

**Published:** 2025-06-25

**Authors:** Jingze Chen, Zhichao Liu, Shujun Zhang, Shuguang Hu, Fazhou Wang

**Affiliations:** ^1^ State Key Laboratory of Silicate Materials for Architectures Wuhan University of Technology Wuhan 430070 China; ^2^ Institute for Superconductor and Electronic Materials Faculty of Engineering and Information Sciences University of Wollongong Wollongong NSW 2500 Australia

**Keywords:** bioinspired materials, carbon mineralized material, mild synthesis, nacre‐like materials

## Abstract

Despite significant advances in the synthesis of biomimetic materials, the scalable fabrication of high‐performance bulk materials under ambient conditions remains a formidable challenge— particularly when it comes to achieving rapid processing while preserving superior mechanical properties. Herein, inspired by nacre's “brick‐and‐mortar” structure, this work develops an energy‐efficient approach that utilizes ice‐templating technology as the structure framework and carbon mineralization for rapid CaCO_3_ production, resulting in a strong yet tough carbon mineralized material (CMM) while also fixing CO_2_. This material replicates both the hierarchical microstructure and chemical composition of nacre. By precisely controlling the freezing dynamics with dual temperature gradients, this work creates an ordered lamellar skeleton composed of γ‐dicalcium silicate (γ‐C_2_S). The rapid, in situ carbon mineralization under mild conditions generates interspersed CaCO_3_ grains within this structure, which are then infiltrated with gelatin to form a nacre‐like CMM with exceptional mechanical properties. The resulting material exhibits a flexural strength of 45 MPa (eight times that of a cement‐hydrogel composite) and a fracture toughness of 2.03 MJ m^−3^ (a 20‐fold improvement over unmodified CMM), while maintaining a density of only 1.2 g cm^−3^. The enhanced performance stems from multiscale toughening mechanisms, including crack deflection, secondary crack formation, and strong interfacial bonding, all of which facilitate efficient energy dissipation. This work establishes a new paradigm for designing high‐performance synthetic materials through the synergistic integration of biomimetic principles with carbon mineralization, offering promising applications in the development of sustainable infrastructure and carbon neutrality.

## Graphical abstract

Inspired by nacre's “brick‐and‐mortar” structure, this work develops an energy‐efficient approach that utilizes ice‐templating technology as the structure framework and carbon mineralization for rapid CaCO_3_ production, resulting in a strong yet tough carbon mineralized material (CMM) under mild conditions while also fixing CO_2_.

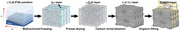

## 1 Introduction

Nature has long been an unparalleled architect of sophisticated materials, crafting structures that combine remarkable mechanical properties with unique functionalities.^[^ ^1^, ^2^ ^]^ Examples include the impact‐resistant dactyl clubs of mantis shrimp,^[^ ^3^ ^]^ the self‐healing properties of bone,^[^ ^4^ ^]^ the adhesive capabilities of gecko feet,^[^ ^5^ ^]^ and the low friction of pitcher plant.^[^ ^6^ ^]^ The synthesis processes, structures, and behaviors of these natural materials and organisms have inspired researchers, leading to the development of advanced functional and engineering materials for diverse applications.^[^ ^7^, ^8^ ^]^

Among various biological materials, nacre, found in mollusk shells, stands out as an exemplary model of structural engineering at the microscale, featuring a relatively simple yet precise structural motif that has proven particularly valuable for designing synthetic materials.^[^ ^9^, ^10^, ^11^ ^]^ This natural composite achieves extraordinary mechanical performance through its unique “brick‐and‐mortar” architecture, wherein CaCO_3_ platelets are meticulously arranged and interconnected by thin organic layers.^[^ ^12^, ^13^ ^]^

Inspired by nacre's structural principles, various synthetic approaches have been developed to fabricate biomimetic materials.^[^ ^11^, ^14^, ^15^ ^]^ Techniques such as vacuum filtration,^[^ ^16^ ^]^ evaporation‐induced self‐assembly^[^ ^17^ ^]^ and spray coating^[^ ^18^ ^]^ have been intensively explored, demonstrating their effectiveness in fabricating nacre‐like films with impressive structures and mechanical properties.^[^ ^19^, ^20^ ^]^ However, these methods are primarily limited to the fabrication of 2D thin films, posing significant challenges in scaling up and producing 3D bulk materials.

Ice‐templating technology has emerged as a promising alternative for creating bulk 3D synthetic materials with directional hierarchical structures by precisely controlling the growth of ice crystal.^[^ ^21^, ^22^, ^23^, ^24^, ^25^, ^26^ ^]^ Researchers have successfully prepared bulk 3D nacre‐like ceramics featuring well‐defined hierarchical structures and excellent mechanical properties.^[^ ^27^, ^28^, ^29^, ^30^ ^]^ However, these materials typically rely on energy‐intensive post‐processing techniques such as hot pressing or sintering for densification. These approaches not only consume a large amount of energy but also hinder the incorporation of temperature‐sensitive functional phases. In contrast, natural biomaterials are synthesized from earth‐abundant elements under ambient conditions,^[^ ^31^ ^]^ suggesting new directions for biomimetic material development. To achieve a more energy efficient fabrication process, the combination of ice‐templating technology with self‐hardening cement‐based materials under mild conditions has proven to be a viable solution.^[^ ^32^, ^33^, ^34^ ^]^ For example, Chen et al. incorporated flexible polyvinyl alcohol (PVA) hydrogels into the rigid lamellar skeleton of the cement paste, resulting in a multilayered cement‐based composite with a remarkable 175‐fold increase in toughness.^[^ ^34^ ^]^ Despite this advancement, the mechanical properties of calcium silicate hydrate (CSH)—the main binding phase in cement hydration—remain limited due to its weak van der Waals and hydrogen bonding, capping the material's flexural strength to merely 5 MPa. Additionally, the inherent complexity of cement hydration complicates the precise microstructural control,^[^ ^35^ ^]^ further limiting the potential applications of cement‐based materials produced using the ice‐templating technique.

Therefore, the current focus is to develop a biomimetic material fabrication strategy that significantly reduces energy consumption during production without sacrificing the mechanical properties. Carbon mineralized material (CMM) provides an ideal platform for the low‐energy fabrication of biomimetic structures, while the inherent design of such structures provides a promising solution to achieving both strength and toughness in CMMs. CMM is a novel material known for its rapid attainment of superior mechanical properties, with CaCO_3_ serving as the structure skeleton and silica gel as the filler phase.^[^ ^36^, ^37^ ^]^ CMM is formed by the reaction of CO_2_ with carbonatable binders, such as silicate minerals and alkali metal oxides, a process that occurs spontaneously and rapidly under ambient temperature and pressure, enabling efficient and eco‐friendly large‐scale production. Despite these advantages, CMM typically possesses large and randomly oriented crystals that lack effective crack deflection and arrest mechanisms,^[^ ^38^, ^39^ ^]^ limiting the simultaneous enhancement of both strength and toughness.^[^ ^40^ ^]^

In this study, we present an innovative approach to prepare unconventional hardening biomimetic composites under mild conditions. By utilizing CO_2_ gas, we transferred the excellent properties of nacres onto the γ‐dicalcium silicate (γ‐C_2_S) carbon mineralization system, creating highly ordered composites with CaCO_3_ as the functional matrix, through dual modulation of both composition and structure. The concept of “mild conditions” involves key parameters such as temperature, pressure, and chemical environment. (i) temperature, the carbon mineralized material can be prepared at ambient temperatures, typically ranging from 0 to 40 °C, without the need for external temperature control. (ii) pressure, the process requires no additional pressure to achieve consolidation. (iii) the chemical environment, the reaction proceeds in an environment of CO_2_, which can be supplied either in high concentrations or from low concentration sources such as industrial fumes. These features distinguish our method from conventional high‐energy fabrication processes and contribute to its energy efficiency and environmental sustainability. We combined ice‐templating technology with carbon mineralization to fabricate nacre‐like hierarchical CMM, offering three distinct advantages over conventional methods: (i) chemical similarity to nacre, due to the CaCO_3_ precipitation during the carbon mineralization process, (ii) precise in situ crystal growth that enables optimized microstructural control, and (iii) processing at ambient temperature and pressure, which reduces energy consumption and allows for the incorporation of diverse functional phases. Leveraging these advantages, we employed ice‐templating technology to create nacre‐like lamellar CMM and then filled the interlayers with organic materials to form a high‐performance organic‐inorganic composite. Through detailed fracture path analysis and finite element simulations, we elucidated the underlying toughening mechanisms and crack deflection behavior, validating our structural design from both microscopic and theoretical perspectives. The resulting nacre‐like hierarchical CMM exhibits remarkable mechanical enhancement, achieving a 2.25‐fold increase in flexural strength and 20‐fold improvement in fracture toughness compared to unmodified CMM. Notably, the material also achieves a low density of 1.2 g cm^−3^, striking an optimal balance between lightweight and high‐strength characteristics. In comparison with cement‐hydrogel composites, our nacre‐like hierarchical CMM demonstrates superior mechanical properties, boasting an 8‐fold increase in flexural strength and double the fracture toughness.^[^ ^34^ ^]^ This work establishes a new paradigm for developing high‐performance materials through the integration of nature‐inspired principles with carbon mineralization technology, paving the way for developing sustainable materials under mild conditions.

## 2 Results

### 2.1 Design and Fabrication Strategy

**Figure 1:**
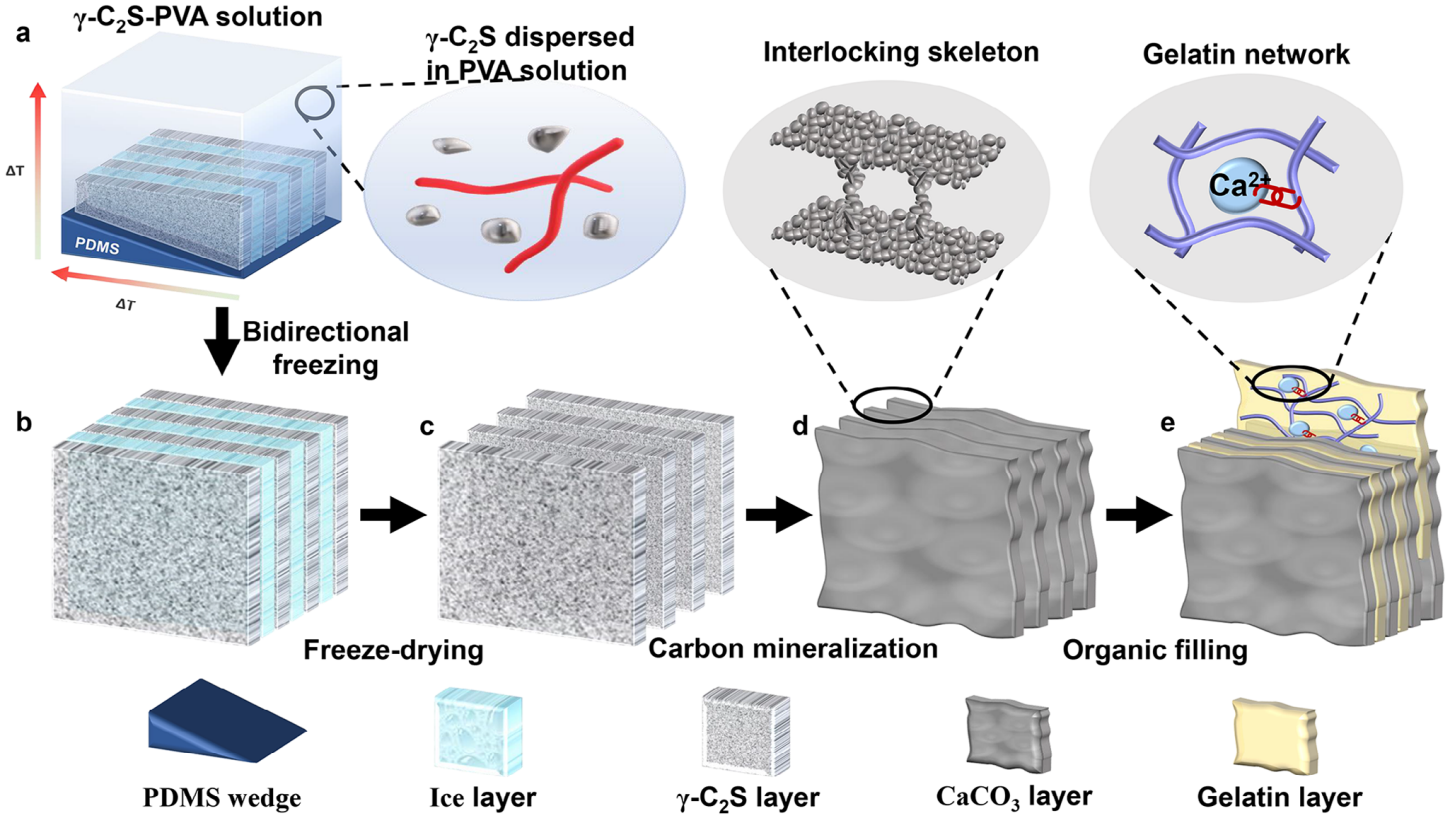
Fabrication of nacre‐like hierarchical CMM. a) The γ‐C_2_S particles are evenly dispersed in PVA solution and poured into the PTFE mold with a PDMS wedge at the bottom, facilitating the directional growth of ice crystals in the slurry under the dual temperature gradients. The inset shows the γ‐C_2_S dispersions in PVA solution. b) Formation of oriented ice‐γ‐C_2_S lamellar structure during freezing. c) Lamellar γ‐C_2_S structure retained after freeze‐drying. d) Conversion to a lamellar CaCO₃ structure via carbon mineralization of γ‐C_2_S. The inset highlights interlocking mineral bridges between CaCO₃ layers. e) Formation of CaCO_3_‐gelatin organic‐inorganic composite by infiltrating gelatin into CaCO_3_ layers. The inset illustrates the gelatin molecular network and Ca^2+^ complexation.

Guided by theoretical principles, we optimized the hierarchical structure and mechanical properties of the nacre‐like hierarchical CMM across molecular, microscopic to macroscopic levels.^[^ ^13^, ^41^ ^]^ The biomimetic mineralization strategy employed γ‐C_2_S as the inorganic structural unit, chosen for its notable facile preparation and exceptional carbon mineralization reactivity, ensuring excellent cementitious properties under ambient conditions. The structural assembly was achieved through a controlled directional freezing process, with PVA serving as the binding agent for γ‐C_2_S particles, which has been demonstrated to be stable in the long term and does not affect the interfacial adhesion and mechanical degradation of the material. Additionally, specific water‐reducing and wetting agents were introduced to reduce the interfacial tension between the liquid and γ‐C_2_S particles, ultimately enhancing the suspension flowability and minimizing particle sedimentation. The dual temperature gradients, vertical (bottom to top) and horizontal (thin to thick), were achieved by a polydimethylsiloxane (PDMS) wedge attached to the copper plate, facilitating the directional growth of ice crystals in the slurry (**Figure** **1**a,b). Following freeze‐drying to achieve an optimal water‐to‐solid ratio (W/S), the hierarchical structure was preserved via in situ carbon mineralization (Figure 1c,d). An appropriate amount of water was a necessary medium for the carbon mineralization reaction, while excess residual ice melting would lead to structural collapse. During the in situ carbon mineralization (Figure S1, Supporting Information), CO_2_ gas dissolved in water, generating H^+^ and CO_3_^2−^ ions. The H^+^ ions facilitated the polarization and subsequent bond cleavage of Ca_2_SiO_4_ to release Ca^2+^ ions, which then combined with CO_3_^2−^ to precipitate as CaCO_3_ (mainly calcite, with a small amount of aragonite and vaterite (Figure S2, Supporting Information)). Concurrently, the residual Si‐O groups underwent hydroxylation followed by dehydration condensation to form SiO_2_ gels. The carbon mineralization reaction yielded a hierarchical CaCO_3_ skeleton within hours, closely resembling the structural features of natural nacre (Figure 1d; Figure S3, Supporting Information). Notably, the mild synthesis conditions, reminiscent of natural biomineralization, provide an ideal building block for biomimetic structural design and functional integration, establishing a versatile platform for advanced materials design.

To optimize the interfacial structure and mechanical properties, biocompatible gelatin was introduced as the interlaminar biopolymer matrix.^[^ ^42^ ^]^ The abundant polar groups in gelatin molecules promote strong interfacial interactions with Ca^2+^ ions at the molecular level. Therefore, the composite was fabricated via vacuum‐assisted infiltration of a low‐viscosity gelatin solution into the interlaminar spaces at 60 °C, followed by controlled drying. This process resulted in an organic‐inorganic composite with a distinct “brick‐and‐mortar” architecture analogous to natural nacre (Figure 1e).

**Figure 2:**
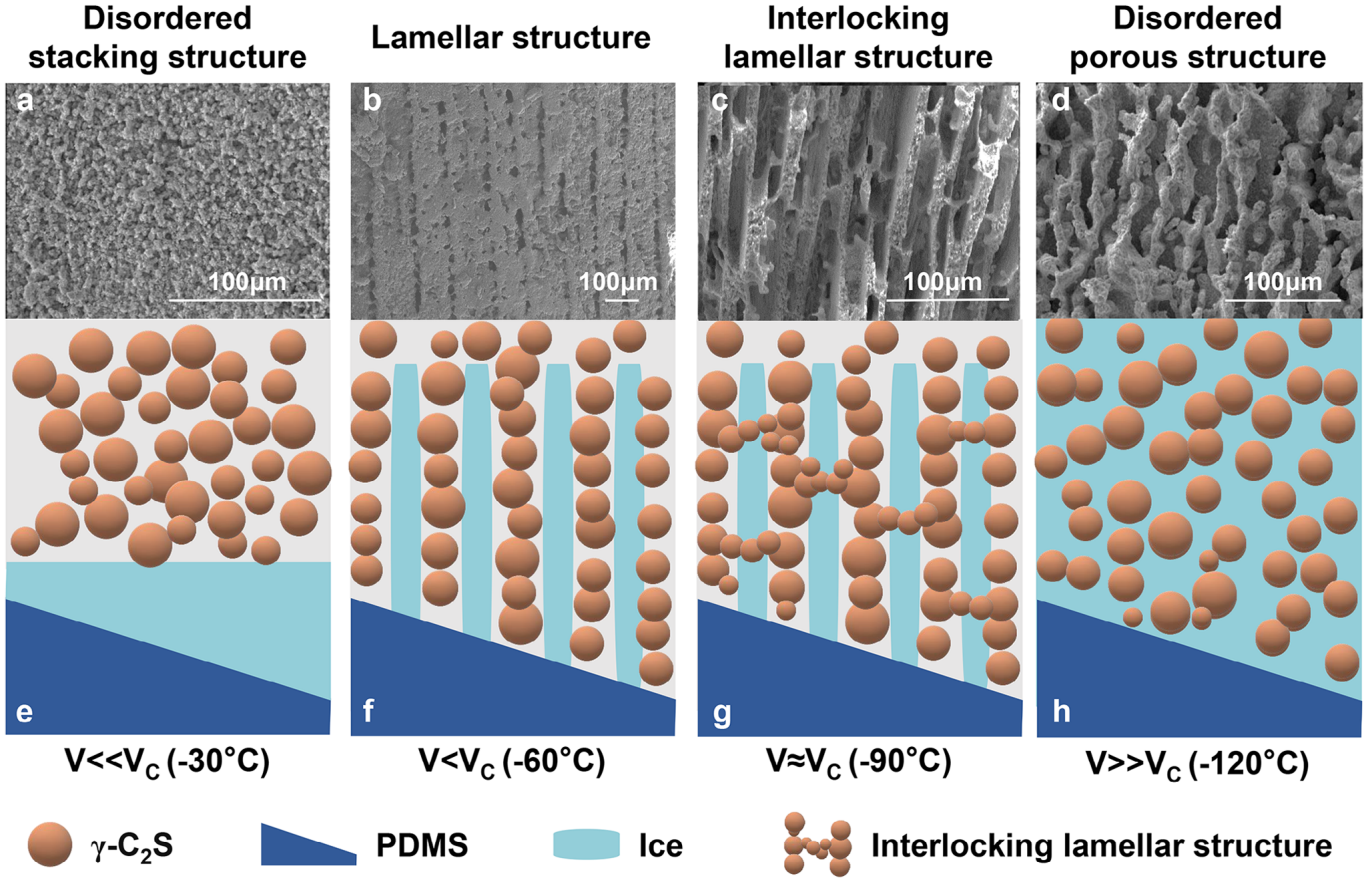
Structural control mechanism of freezing dynamics. a–d) SEM images of microstructural features of CMMs at different freezing rates. e–h) Schematic of freezing front progression at different freezing rates.

To elucidate the structural evolution mechanisms during freezing, we systematically investigated the relationship between freezing dynamics and microstructural evolution by introducing a critical freezing rate (Vc).^[^ ^22^ ^]^ At extremely low freezing rates (V<<Vc), ice crystals advanced as a planar front along the PDMS mold surface, causing γ‐C_2_S particles to concentrate in the unfrozen regions (**Figure** **2**e). The slow growth of the ice crystals allowed sufficient time for the rearrangement of γ‐C_2_S particles, resulting in their random packing (Figure 2a). As freezing rate increased (V<Vc), the accelerated growth of ice crystals expelled γ‐C_2_S particles to the boundaries of the crystals, forming ordered lamellar structures guided by the dual temperature gradients (Figure 2b,f). At the critical freezing rate (V ≈ Vc), the rapid growth of ice crystals partially entrapped γ‐C_2_S particle, creating mineral bridges between adjacent layers and forming an interlocking lamellar structure (Figure 2c,g). At higher freezing rates (V>>Vc), the rapid solidification of the ice crystals prevented particle migration, resulting in γ‐C_2_S particles being immobilized, forming disordered porous structures (Figure 2d,h). This freezing dynamics‐based structural control strategy provides crucial insights for optimizing material fabrication processes.

### 2.2 Fabrication of Nacre‐Like Hierarchical CMM: Microstructure and its Relationship With Mechanical Properties

**Figure 3:**
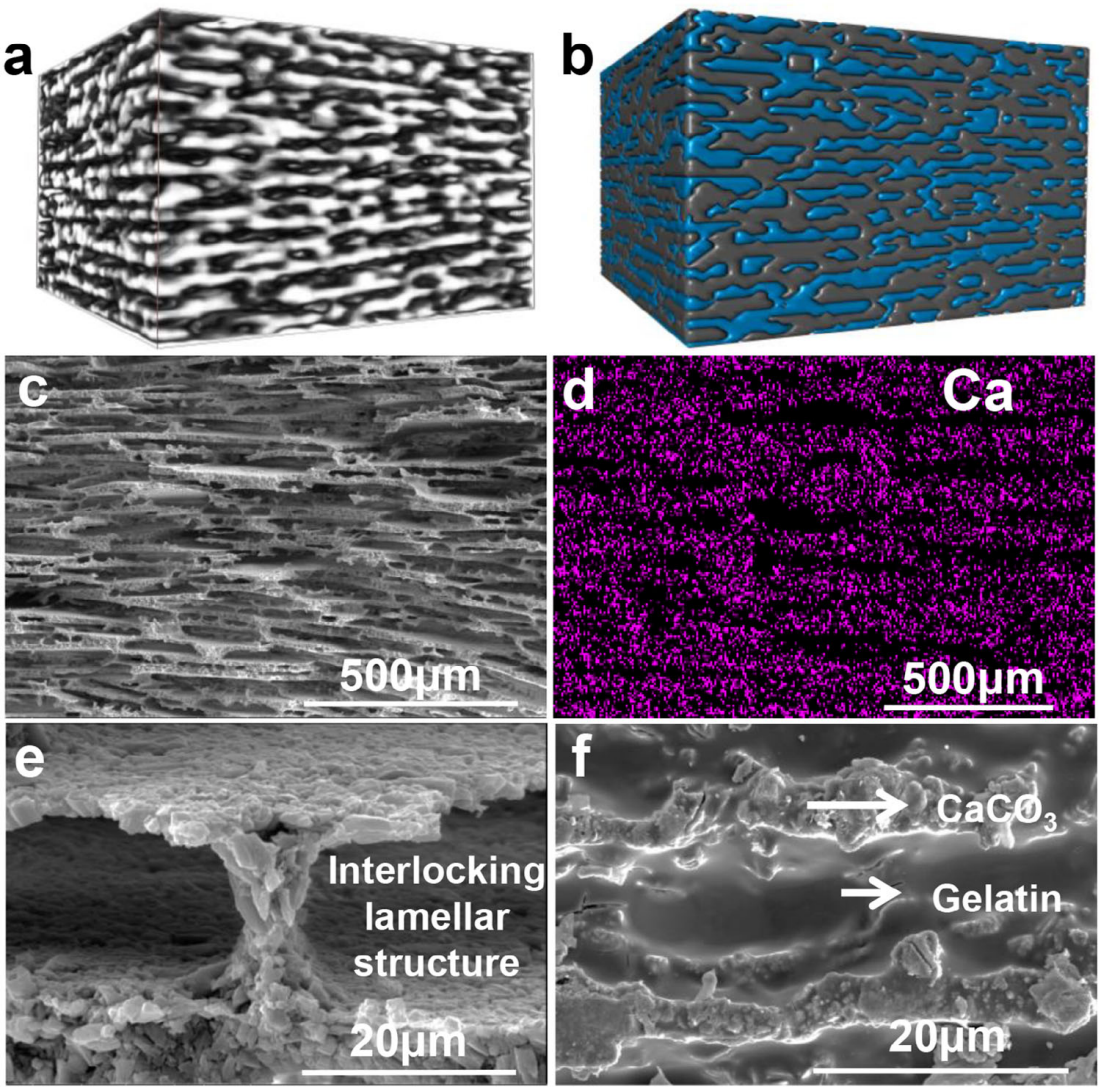
Structural characteristics of nacre‐like hierarchical CMM at different scales. a,d) 3D micro‐computed tomography (µ‐CT), revealing their highly oriented structure. b) µ‐CT of the final composite structure, demonstrating the preserved lamellar architecture after gelatin impregnation. c) SEM images of the CMM's lamellar skeletons d) EDS mapping of Ca distribution in the CMM, highlighting the uniform lamellar organization. e) High‐magnification SEM image showing the interlocking structure between CaCO_3_ layers, crucial for the enhanced mechanical performance. f) Detailed microstructure of the composite, illustrating the well‐defined organic‐inorganic interface between CaCO₃ and the gelatin matrix.

The sophisticated hierarchical architecture and the synergy between its organic‐inorganic interface are the primary mechanisms underlying the superior mechanical properties of natural nacre, providing fundamental inspiration for our biomimetic design. As shown in **Figure** **3**, the biomimetic CMM with a hierarchical structure spanning multiple length scales was produced using ice‐templating technology. At the mesoscopic level, 3D micro‐computed tomography (µ‐CT) revealed continuous and ordered lamellar architectures with distinct anisotropic orientations (Figure 3a,b), corroborated by elemental mapping of Ca distribution via EDS (Figure 3d). SEM images (Figure 3c) demonstrated uniform interlayer spacing (IS) and highly ordered lamellar arrangements. At the microscopic scale, nano‐sized CaCO_3_ crystals self‐assembled into regular hierarchical structures along the ice crystal growth direction, as shown in Figure 3e, where mineral bridges and interlocking lamellar structures were identified as critical features contributing to the enhanced mechanical properties.

The vacuum‐thermal assisted infiltration technology facilitated effective organic‐inorganic integration. By controlling the vacuum conditions and temperature fields, interlayer air and capillary resistance were significantly reduced, allowing the gelatin solution to overcome surface tension and viscosity limitations for rapid, uniform infiltration between CaCO₃ layers, as confirmed by detailed SEM analysis (Figure 3f). The infiltration process ensured complete coverage of the interlayer organic phase and improved interfacial adhesion, creating stable organic‐inorganic interfaces. This multi‐scale structural design, achieved under mild conditions successfully replicated the hierarchical structures of natural biological materials, yielding nacre‐like hierarchical CMM with mechanical properties comparable to those of natural nacre as well as those processed under harsh conditions.

**Figure 4:**
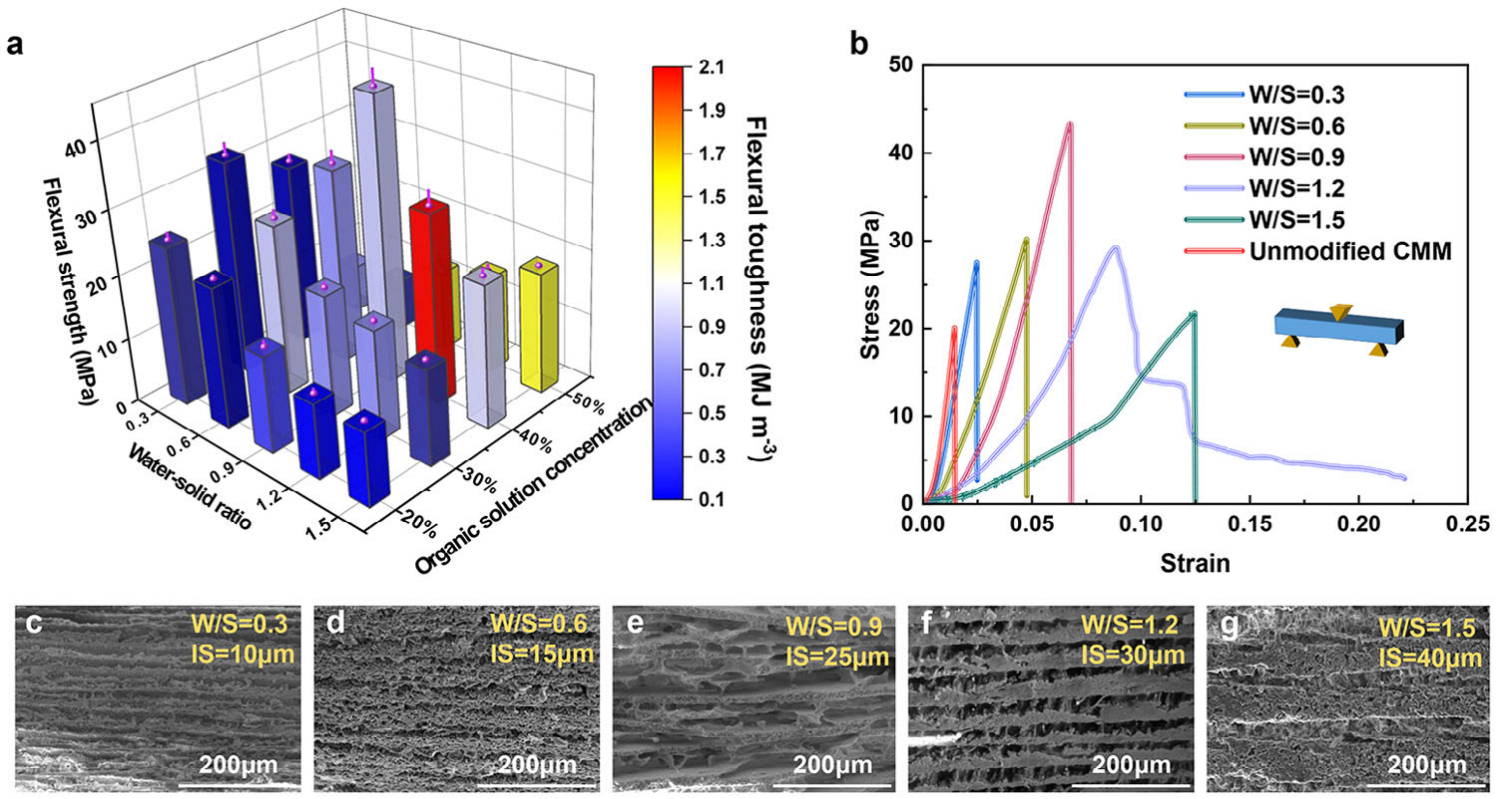
Mechanical properties and microstructures of nacre‐like hierarchical CMMs. a) The effect of W/S and organic solution concentration on flexural strength and flexural toughness of CMM. b) Flexural stress–strain curves of the CMMs fabricated with different W/S and molding processes. c–g) SEM images of the microstructures of CMMs fabricated with different W/S. The error bars represent the s.d. of at least five replicate measurements.

To optimize the fabrication protocol of nacre‐like hierarchical CMM, we systematically investigated the effects of two critical processing parameters, specifically the W/S and gelatin solution concentration, on the mechanical properties. Through orthogonal experimental design focusing on flexural strength and flexural toughness, we identified optimal mechanical properties at W/S = 0.9 and 40 wt% gelatin solution, achieving a flexural strength of 45 MPa, representing a 125% enhancement over unmodified CMM (**Figure** **4**a,b). Microstructural analysis via SEM revealed that increasing W/S from 0.3 to 1.5 resulted in a systematic expansion of interlayer spacing from 10 to 40 µm (Figure 4c–g). This structural evolution was manifested in the stress‐strain response as a progressive reduction in the elastic modulus as the W/S ratio increased (Figure 4b). This decrease was primarily attributed to a lower material density and reduced inorganic mineral content, both of which contributed to a reduction in overall material stiffness. Additionally, the fracture toughness measurements peaked at 2.03 MJ m^−^^3^ when the W/S ratio was 1.2 and the gelatin solution concentration was 40%, marking a 20‐fold enhancement compared to unmodified CMM (Figure 4a,b). This exceptional improvement can be attributed to the formation of well‐defined lamellar architectures at higher W/S ratios, coupled with optimal interlayer infiltration facilitated by the 40 wt% gelatin solution. These factors contributed to a superior energy dissipation, significantly improving mechanical performance.

**Figure 5:**
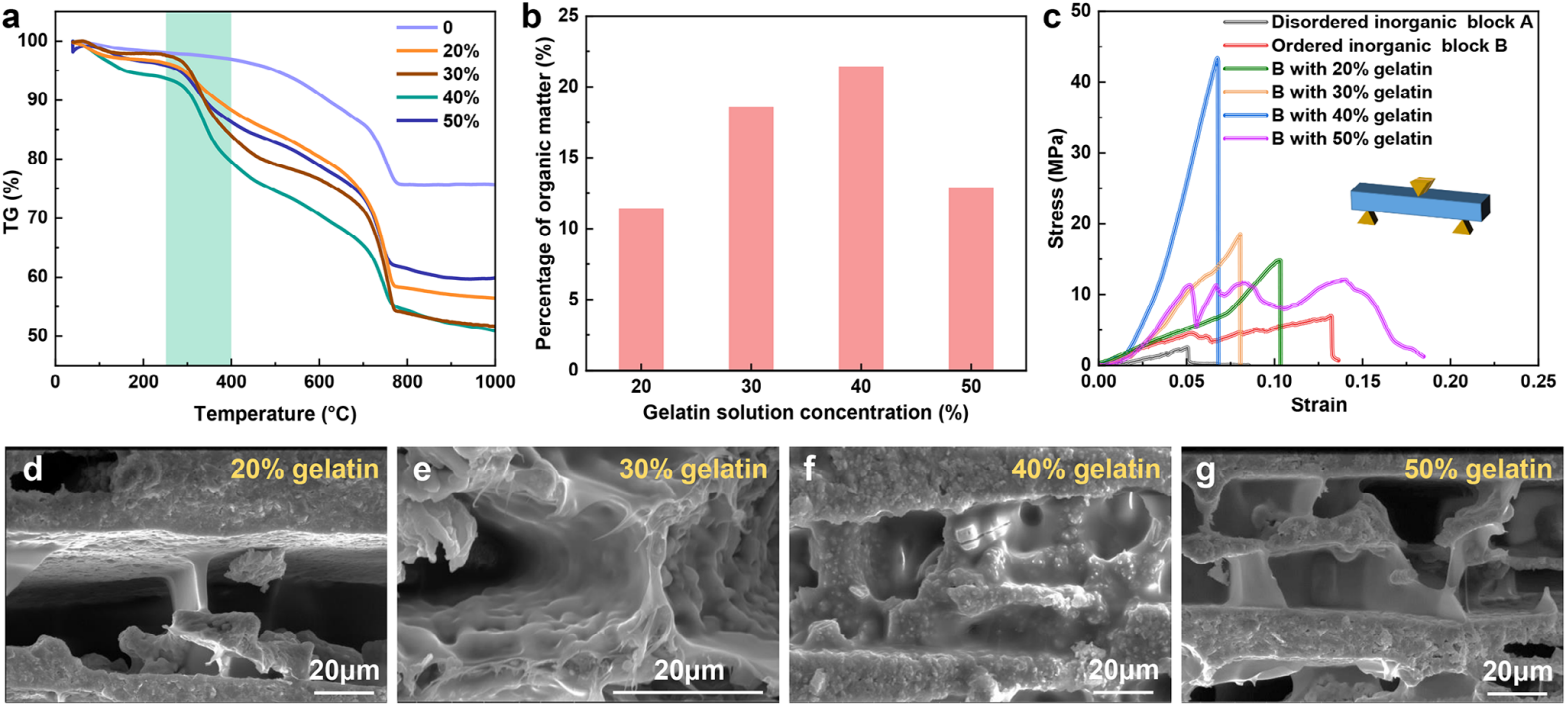
Mechanical properties and compositions of nacre‐like hierarchical CMMs. a) TG curves of CMMs fabricated with different gelatin solution concentrations. b) Organic content quantification in CMMs versus gelatin concentration. c) Flexural stress–strain curves of the SMMs fabricated with different organic solution concentrations. d–g) Microstructural variations with increasing organic concentration.

To explore the impact of the organic phase on the composite's performance, thermogravimetric analysis (TGA) was conducted to quantify the composition. As shown in **Figure** **5**a, the weight loss observed between 250–400 °C, corresponding to gelatin decomposition, indicated that 40 wt% gelatin solution achieved optimal infiltration, resulting in an organic content of 21.4 wt% (Figure 5b). This composition closely matched the mechanical performance observed in testing. Stress‐strain curves revealed that the elastic modulus increased progressively with gelatin concentration in the range of 20–40 wt% (Figure 5c). This enhancement was attributed to the formation of more continuous and denser organic networks between the mineral layers, as confirmed by SEM observations (Figure 5d–f).

### 2.3 Mechanical Properties Comparison: Nacre‐Like Hierarchical CMM and its Counterparts

**Figure 6:**
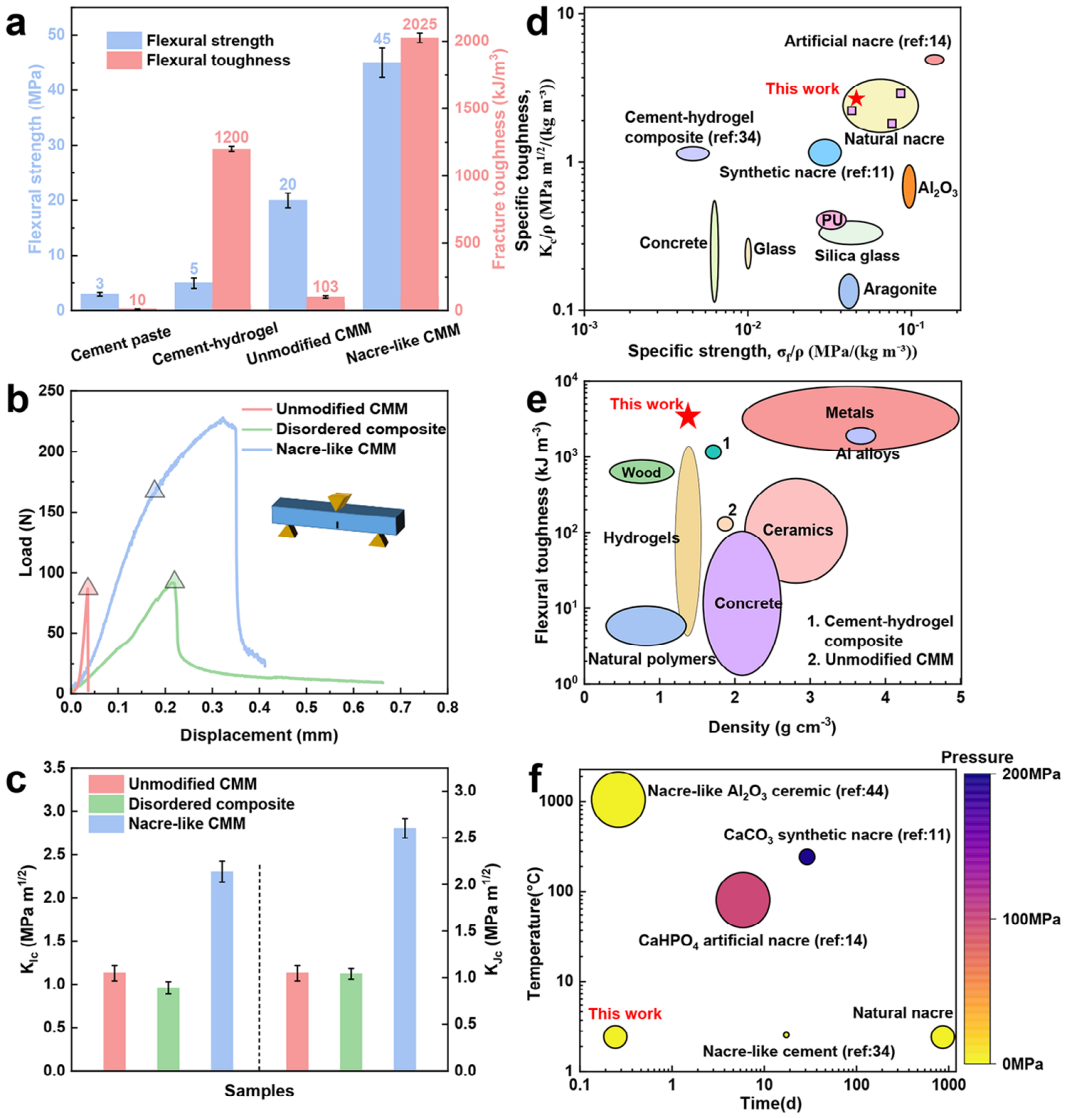
Mechanical properties of nacre‐like hierarchical CMM compared with various natural and synthetic materials. a) Flexural strength and flexural toughness of nacre‐like hierarchical CMM compared with cement paste, cement‐hydrogel composite, and unmodified CMM. b) Flexural stress–strain curves of CMMs using the SENB method. c) Fracture toughness for crack initiation (K_Ic_) and stable crack propagation (K_Jc_) of nacre‐like hierarchical CMM compared to unmodified CMM and disordered composite. Ashby diagram of specific strength versus specific toughness d) and flexural toughness versus density.^[^ ^11^, ^14^, ^34^ ^]^ e) The studied nacre‐like hierarchical CMM compared with a range of natural and synthetic materials.^[^ ^34^, ^43^ ^]^ f) Ashby diagram of time versus temperature versus pressure versus specific strength for the studied nacre‐like hierarchical CMM compared with their counterparts, where the circle size in the figure represents the magnitude of the specific strength value.^[^ ^11^, ^14^, ^34^, ^44^ ^]^ The error bars represent the s.d. of at least five replicate measurements.

A comprehensive mechanical performance evaluation highlights the distinctive advantages of the studied nacre‐like hierarchical CMM through systematic comparisons with pure cement paste, ice‐templating cement‐hydrogel composite, and unmodified CMM (**Figure** **6**a). The nacre‐like hierarchical CMM exhibits a synergistic enhancement in strength and toughness, achieving a flexural strength of 45 MPa, nine times that of the cement‐hydrogel composite (5 MPa).^[^ ^34^ ^]^ Contrary to cement‐based materials, which also exhibit self‐hardening properties under mild conditions, the superior performance of CMM can be attributed to the compositional similarity to natural nacre (CaCO_3_ and organic matrix), which offers inherent advantages over the CSH units found in conventional cement‐based materials, particularly in biomimetic structural design.

To further investigate the toughening effect, we evaluated fracture behavior using the single‐edge notched beam (SENB) method^[^ ^45^ ^]^ with the stress‐strain curve shown in Figure 6b. Key metrics, including resistance to crack initiation (K_Ic_) and resistance to crack propagation during plastic deformation (K_Jc_),^[^ ^11^ ^]^ were evaluated. As shown in Figure 6c, the nacre‐like hierarchical CMM achieved a K_Ic_ value of 2.3, substantially exceeding of the values for unmodified CMM (1.13) and disordered composite (0.96). Notably, the nacre‐like hierarchical CMM exhibited unique progressive failure characteristics, undergoing extensive plastic deformation after elastic deformation rather than catastrophic failure. This behavior elevates the K_Jc_ value of nacre‐like hierarchical CMM to 2.8, while unmodified CMM with negligible plastic deformation, maintains a value essentially identical to its K_Ic_.

Further comparisons of the specific toughness, specific strength, fracture energy, and density with various natural and synthetic materials demonstrate that our nacre‐like hierarchical CMM rivals the performance metrics of natural nacre, significantly surpassing conventional cement, glass, organic materials, and pure aragonite (Figure 6d). Meanwhile, as a novel lightweight, high‐strength, high‐toughness material, its density is notably lower than that of all cement‐based materials, including ice‐templating cement‐hydrogel composites,^[^ ^34^ ^]^ further emphasizing its superior specific toughness (Figure 6e). Additionally, CMM exhibits excellent durability, remaining intact after 10000 cyclic loading tests (Figure S4, Supporting Information). Furthermore, we systematically compared the processing time, temperature, pressure, and mechanical properties of various materials, as shown in Figure 6f. While natural nacre, cement‐hydrogel composites, and ceramics can be prepared at atmospheric pressure, they are limited by long formation times (10^3^ d), poor mechanical properties (5 MPa) or high sintering temperatures (1000–1400 (C), respectively. Other artificial nacres, constrained by high‐temperature and high‐pressure hot‐pressing processes (100 (C and 100 MPa), lack scalability. In contrast, our CMM can be rapidly fabricated at mild temperature and ambient pressure without any additional energy consumption during the curing process, while achieving mechanical properties comparable to natural nacres and bioinspired materials that typically require harsh processing conditions. Moreover, the mild fabrication process avoids the degradation of temperature‐sensitive functional components, such as biomolecules (e.g., enzymes, proteins, and drug carriers) and metallic phases (e.g., conductive metals and magnetic alloys), which are typically compromised during high‐temperature sintering. This enables the integration of a wider range of functional materials in the design. This remarkable achievement challenges the conventional belief that high‐performance structural materials must involve energy‐intensive processing, simultaneously advancing structural engineering capabilities and sustainability goals.

### 2.4 Underlying Mechanism: Toughening Mechanisms Analysis

**Figure 7:**
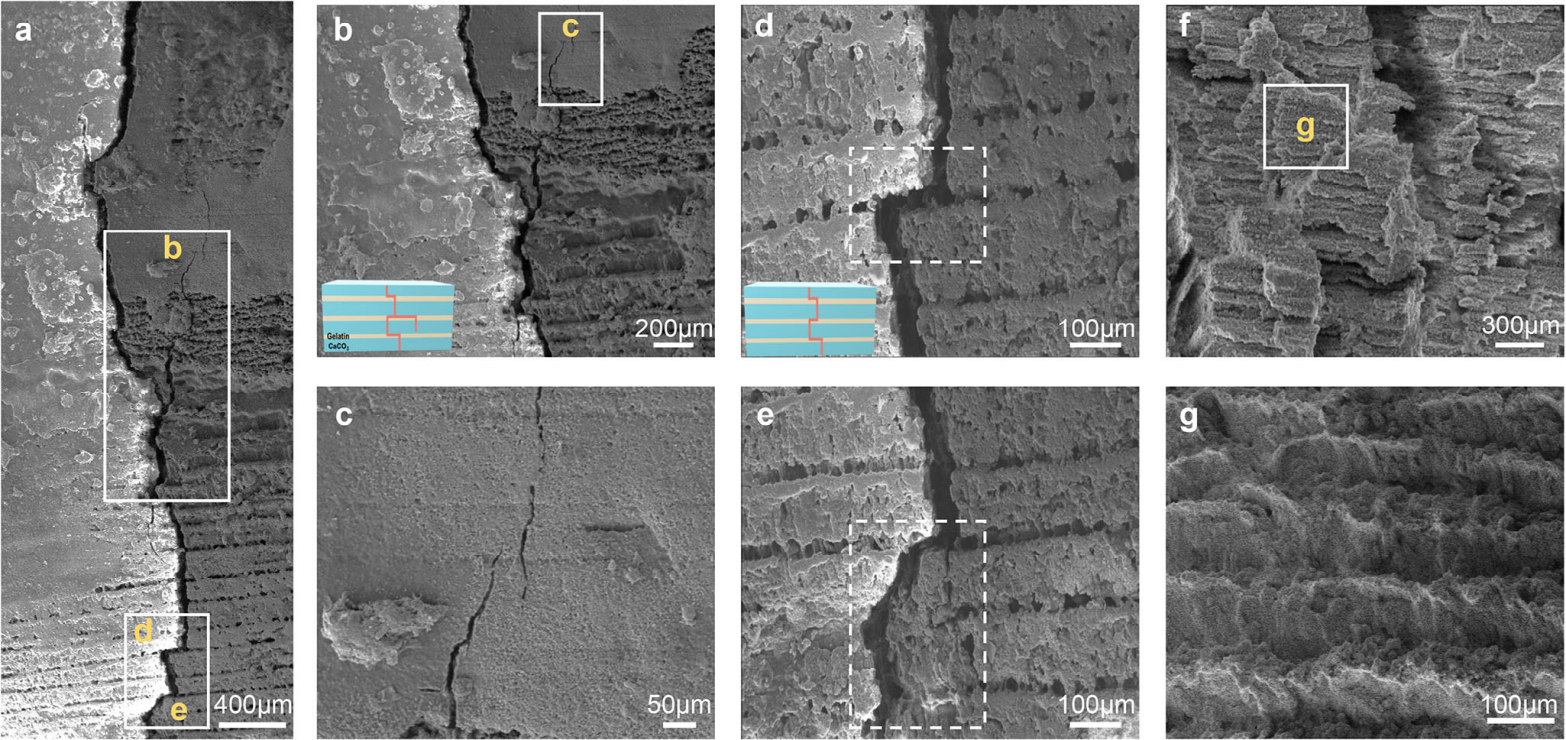
Multiple toughening mechanisms based on crack deflection acting at multiple length scales. a) Long‐range crack deflection. b) Formation of new secondary cracks during primary crack propagation. The inset shows the toughening mechanism based on new crack sprouting. c) Crack bridging at the crack tips. d,e) Cack deflection and expansion along the interlayer. The inset shows the toughening mechanism based on the transverse deflection of cracks along the interlayer. f,g) Fracture surfaces display regular “brick‐and‐mortar” structures.

The multi‐scale toughening mechanisms in the nacre‐like hierarchical CMM were elucidated through detailed fracture behavior analysis and theoretical simulations. As shown in **Figure** **7**a, cracks propagated along tortuous paths until the material was fully penetrated and failed, a primary toughening mechanism reminiscent of natural nacre.^[^ ^23^ ^]^ High‐magnification SEM images revealed extensive secondary crack formation during primary crack propagation (Figure 7b), accompanied by pronounced crack bridging at the crack tips (Figure 7c). The features collectively contributed to substantial energy dissipation.^[^ ^40^, ^46^ ^]^ Furthermore, the gelatin's lower modulus and higher Poisson's ratio compared to CaCO_3_ induced shear sliding, plastic deformation, and localized fracture within the organic phases. These mechanisms facilitated crack deflection and energy dissipation along the interlayers (Figure 7d,e). SEM images of the fracture surfaces (Figure 7f,g) further confirmed the preservation of the regular “brick‐and‐mortar” structure, which closely resembled that of natural nacre.

**Figure 8:**
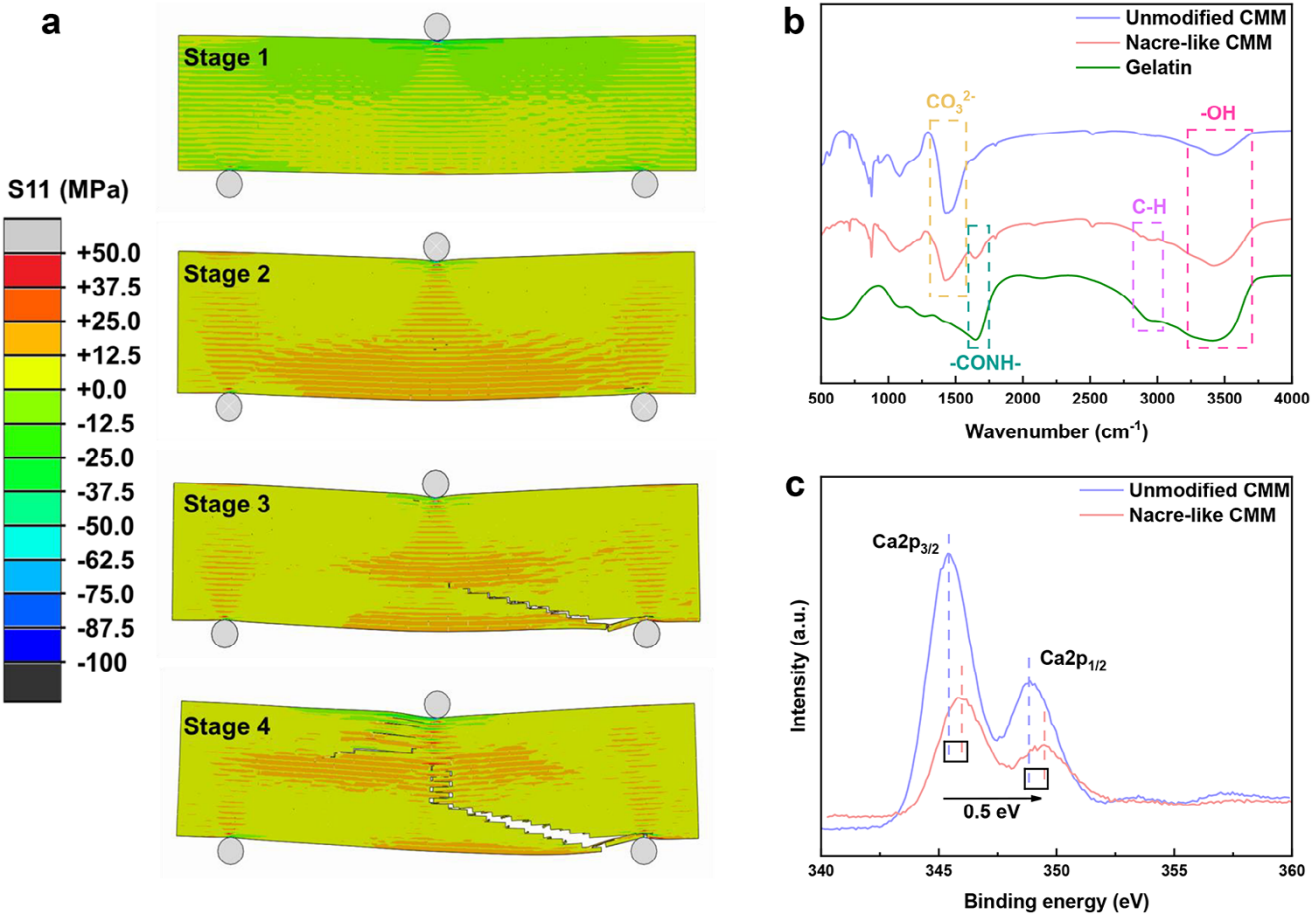
Theoretical simulations and experimental characterizations of the nacre‐like hierarchical CMM. a) The internal stress distribution and crack propagation of nacre‐like hierarchical CMM in a three‐point bending experiment via nonlinear finite element model simulation. FTIR b) and XPS c) patterns of the nacre‐like hierarchical CMM, unmodified CMM, and gelatin.

Moreover, finite element analysis (**Figure** **8**a) provides additional insights into the stress distribution and crack propagation mechanisms within the nacre‐like hierarchical CMM. Under three‐point bending loading conditions, the biomimetic architecture facilitated a notably uniform stress distribution across the matrix, effectively mitigating crack initiation that typically arises from stress concentrations (Stages 1 and 2). As cracks propagated, the organic‐inorganic composites demonstrated characteristic fracture behaviors, including microcrack formation and crack deflection along interlayers (Stages 3 and 4). These synergistic mechanisms substantially enhanced the material's fracture resistance. The theoretical simulations showed remarkable consistency with experimental stress‐strain responses and microstructural observations, further validating our biomimetic design strategy and confirming the successful replication of natural nacre's mechanical behavior in our nacre‐like hierarchical CMM.

To gain a deeper understanding of the chemical binding mechanism at the organic‐inorganic phase interface, we systematically analyzed the intermolecular forces and chemical interactions in unmodified CMM, nacre‐like hierarchical CMM, and gelatin by Fourier transform infrared spectroscopy (FTIR), X‐ray photoelectron spectroscopy (XPS) and zeta potential. As shown in Figure 8b, the absorption peak at 1435 cm^−1^ was attributed to the vibration of CO_3_^2−^, which also appeared in the nacre‐like hierarchical CMM and was shifted toward the lower wavelengths down to 1420 cm^−1^, indicating strong intermolecular interactions at the interface of CaCO_3_ and gelatin. The characteristic absorption peak of the ‐CONH‐ group in the gelatin molecule shifted from 1652 to 1642 cm^−1^, revealing the formation of a stable coordination between the polar amide group and Ca^2+^ ions. In addition, the vibrational absorption peaks of C‐H bonds observed at 2900 cm^−1^ remained clearly visible in the nacre‐like hierarchical CMM, further confirming the effective dispersion of the organic phase in the CaCO_3_ matrix and the excellent interfacial compatibility. The XPS analysis (Figure 8c) provided further evidence of these interfacial interactions. The increased Ca 2p binding energies in the nacre‐like hierarchical CMM compared to unmodified CMM suggested significant coordination between gelatin molecules and Ca^2^⁺ ions. Moreover, this coordination altered the chemical environment of the Ca atoms, as evidenced by the broadening of the half‐height width in the XPS spectrum (2.45–2.61 eV).^[^ ^47^ ^]^ The adsorption of polar groups onto the CaCO₃ surface partially obscured the surface Ca atoms, leading to a reduction in the intensity of the Ca 2p peak.^[^ ^47^ ^]^ We also tested the zeta potential of CaCO_3_ and gelatin to quantify the interfacial adhesion strength. As shown in the Figure S5 (Supporting Information), CaCO_3_ is positively charged (+5 mV) due to the exposure of calcium ions on the surface of CaCO_3_ as a result of the continuous dissolution of CO_2_ during the reaction (pH < isoelectric point), whereas gelatin is negatively charged (−18 mV) due to the presence of carboxyl groups, resulting in a strong interfacial adhesion strength between them. These analyses collectively confirmed the formation of stable chemical bonds between CaCO₃ and gelatin at the molecular level, offering a microscopic explanation for the exceptional mechanical properties observed in the nacre‐like hierarchical CMM.

## 3 Conclusion 

This work marks a major step in the field of sustainable and high‐performance materials by successfully replicating the natural structure of nacre in bulk carbon mineralized materials. By combining ice‐templating technology with carbon mineralization, we achieve precise structural control across multiple length scales. The resulting materials exhibit mechanical properties comparable to natural nacre, including a remarkable strength of 45 MPa and toughness of 2.03 MJ m^−^^3^, while maintaining practical advantages such as reduced density and the ability to be process under mild conditions. The proposed approach eliminates the need for energy‐intensive processing methods typically required for similar materials, aligning with sustainability goals. Additionally, the detailed analysis of toughening mechanisms and the optimization of processing parameters provide valuable insights for the design of next‐generation bio‐inspired materials. These findings establish a new paradigm for creating environmentally friendly synthetic materials with superior mechanical properties, offering transformative potential for sustainable infrastructure development.

## 4 Experimental Section

### Synthesis of γ‐C_2_S Powders

Stoichiometric quantities of Ca(OH)_2_ and SiO_2_ (2:1 molar ratio) were combined with an equal mass of deionized water and then ball‐milled at 300 rpm for 2 h. The resulting mixture was dried at 105 °C for 12 h and then subjected to calcination at 1400 °C for 3 h. The calcined material was allowed to cool in a furnace, yielding γ‐C₂S powders.

### Preparation of γ‐C_2_S Suspensions

γ‐C_2_S suspensions were prepared by mixing 100 g γ‐C_2_S powder with deionized water at varying W/S (0.3, 0.6, 0.9, 1.2, 1.5). 1 g polycarboxylic acid water‐reducing agent was added as a dispersant and lubricant to enhance the flowability of the slurry. 0.1 g alkyne glycol ethoxylate was added as a wetting agent to reduce the interfacial tension between the liquid and γ‐C_2_S powders. 0.5 g PVA was incorporated as a binder to prevent particle sedimentation during freezing and structural collapse during freeze‐drying. The mixture was homogenized and then processed in a mixer at 1000 rpm for 10 min.

### Fabrication of Carbon Mineralized Materials With Lamellar Skeletons

The γ‐C_2_S suspension was poured into a 5 × 10 × 20 mm PTFE mold with a PDMS wedge (20° angle) at the bottom. Bidirectional temperature gradients were established in both vertical and horizontal directions to precisely control ice crystal growth orientation using a copper plate‐liquid nitrogen assembly. The bottom of the mold was in contact with a copper plate which was connected to a liquid nitrogen chamber for efficient temperature transfer. After 1 h of freezing, the frozen specimens were rapidly demolded and vacuum freeze‐dried at −80 (C until reaching a W/S of 0.2. The specimens were placed in stainless steel pressure tank, followed by vacuuming and introduction of 99.99% purity CO_2_ gas for 4 h, eventually yielding inorganic specimens with lamellar calcium carbonate skeletons.

### Fabrication of Nacre‐like Hierarchical Carbon Mineralized Materials

Gelatin solutions with varying concentrations (20 wt%, 30 wt%, 40 wt%, 50 wt%) were prepared for vacuum impregnation of the lamellar CMM at 60 °C in a vacuum oven. Multiple impregnation cycles were performed until no obvious bubbles were observed in the solution. Finally, nacre‐like hierarchical CMM were obtained.

### Sample Characterizations

Fourier transform infrared spectroscopy (FTIR, Nicolet 6700) was carried out over 400–4000 cm‐1 at room temperature. X‐ray photoelectron spectroscopy (XPS, ESCALAB 250Xi) with a scanning stepwise of 0.1 eV was performed to probe the binding energies of Ca. X‐ray diffraction (XRD) measurements were conducted using a Malvern Panalytical diffractometer (CuKα radiation, λ = 0.15 406 nm). Data acquisition spanned a 2θ range of 10°–80° with a step size of 0.02° and a counting time of 2 s per step. Quantitative phase analysis was performed via the Rietveld refinement method using HighScore Plus software, with 10 wt% α‐Al₂O₃ (corundum) added as an internal standard for absolute phase quantification. The zeta potential was tested using a nanoparticle size potentiostat model Nano ZS ZEN3600 zetasizer (Malvern).

### Microstructure Characterizations

The microstructure and fracture pathways of the composites were visualized using scanning electron microscopy (SEM, FEI QUANTA FEG 450 ESEM with a BSE detector) at an acceleration voltage of 5 kV. The distribution characteristics of Ca elements along the fracture surface were characterized using energy dispersive spectroscopy (EDS). The internal structure of the composites was visualized using micro‐computed tomography (µ‐CT, ZEISS Xradia 510 Versa, 80 kV acceleration voltage), and subsequent analysis of the data was carried out using Dragonfly software.

### Mechanical Properties

Flexural strength was evaluated through three‐point bending tests on beam specimens (20 mm × 10 mm × 5 mm) using an electro‐hydraulic servo testing system (SHT4106, 1000 kN capacity). Tests were conducted with an 18 mm support span at 0.002 mm·s^−1^ displacement rate. Stress‐strain relationships were analyzed and fracture energy was calculated as the integrated area under these curves. For the single edge notched bend (SENB) test, a 200 µm thick diamond blade was used to notch the sample to ≈50% of its thickness, and the remaining procedure was identical to the three‐point bending tests. All mechanical data represent averages from five independent specimens. The fatigue test was performed using 60% of the maximum stress of the material during cyclic loading with 10000 cycles at a frequency of 2 Hz.

### Calculation of Mechanical Properties—Fracture Toughness, *K_Jc_*, is Determined by

(1) $$K_{\mathrm{Jc}}=\sqrt{\left(J_{\mathrm{el}}+J_{\mathrm{pl}}\right)E^{\prime }}$$

where *J_el_* is the elastic component, *J_pl_* is the plastic component and *E*′ is given by

(2) $$E^{\prime }=\frac{E}{1-v^{2}}$$

where *E* is the elastic modulus and *v* is the Poisson's ratio. Since the effect of *E* on *K_Jc_* is minimal, *E*′ can be approximated as E.

### Calculation of Mechanical Properties—Elastic component, *J_el_*, is Determined by

(3) $$J_{\mathrm{el}}=\frac{K_{\mathrm{Ic}}^{2}}{E^{\prime }}$$

### Calculation of Mechanical Properties—Fracture toughness, *K_Ic_*, is Determined by

(4) $$K_{\mathrm{Ic}}=\frac{PS}{BW^{3/2}}f\left(a/W\right)$$

where *P* is the load, *S* is the span, *B* and *W* are the width and thickness of the specimen, *a* is the initial crack length also known as the notch depth. The function *f* is given by

(5) $$\begin{array}{ccc} & & f\left(a/W\right)=\\ & & \frac{3{\left(a/W\right)}^{1/2}\left[1.99-\left(a/W\right)\left(1-a/W\right)\left(2.15-3.93a/W\right)+2.7{\left(a/W\right)}^{2}\right]}{2\left(1+2a/W\right){\left(1-a/W\right)}^{3/2}} \end{array}$$

### Calculation of Mechanical Properties—The plastic Component, *J_pl_*, is Calculated by

(6) $$J_{\mathrm{pl}}=\frac{2A_{\mathrm{pl}}}{Bb}$$

where *A_pl_* represents the plastic region area under load‐displacement curve and *b* is the remaining ligament of the crack.

### Finite Element Method Simulation

The CaCO_3_‐gelatin layered composite with a “brick‐and‐mortar” architecture was systematically investigated through finite element analysis using ABAQUS software. The model replicated experimental conditions with specimen dimensions of 20 mm × 5 mm × 1 mm under three‐point bending (16 mm span). The numerical model consisted of alternating rigid (CaCO_3_) and soft (gelatin) layers. A thickness ratio of 4:1 between CaCO_3_ and gelatin layers was confirmed by SEM analysis, with cohesive elements embedded at interfaces to simulate crack behavior (Figure S3, Supporting Information). We implemented two distinct cohesive formulations to characterize the mechanical response at different interfaces: those between adjacent CaCO_3_ grains, and those at the CaCO_3_‐gelatin boundaries. The interfacial damage behavior was governed by a maximum stress criterion‐based traction‐separation law. The elastic modulus and Poisson's ratio were set as 76.8 GPa and 0.32 for CaCO_3_ layers, and 3 GPa and 0.48 for gelatin layers, respectively. Zero‐thickness cohesive elements with a critical displacement of 0.01 mm were employed to model interfacial failure. A user‐defined subroutine (VUSFLD) was integrated to capture the progressive damage evolution in gelatin layers based on maximum principal strain criteria.

## Conflict of Interest

The authors declare no conflict of interest.

## Author contributions

J.Z.C. and Z.‐C.L. designed the experiments. F.‐Z.W., S.‐J.Z., and S.‐G.H. supervised the research. J.‐Z.C. conducted the experiments, J.‐Z.C. designed the figures and the Supplementary Information while Z.‐C.L. and J.‐Z.C. wrote the main paper. S.‐J.Z. revised the paper. All authors discussed the results, and their implications and revised the manuscript and figures at all stages.

## Supporting information



Supporting Information

Supporting Information

## Data Availability

The data that support the findings of this study are available from the corresponding author upon reasonable request.
