# Supplementary material for: Energy‐Efficient Fabrication of Biomimetic Materials for Sustainable Infrastructure Applications
Source: Adv Sci (Weinh). 2025 Jun 25;12(35):e03854. doi: 10.1002/advs.202503854 (PMC12463093; doi:10.1002/advs.202503854)
Supplement: Supplementary file 1 — Supporting Information [file ADVS-12-e03854-s001.docx]

**Energy-Efficient Fabrication of Biomimetic Materials for Sustainable Infrastructure Applications**

Jingze Chen, Zhichao Liu^*^, Shujun Zhang^*^, Shuguang Hu, Fazhou Wang^*^

J. Chen, Z. Liu, S. Hu, F. Wang

State Key Laboratory of Silicate Materials for Architectures

Wuhan University of Technology, Wuhan, 430070, China

S. Zhang

Institute for Superconductor and Electronic Materials, Faculty of Engineering and Information Sciences

University of Wollongong, Wollongong, NSW, 2500, Australia

Corresponding author:

Zhichao Liu, [liuzc9@whut.edu.cn](mailto:liuzc9@whut.edu.cn), Shujun Zhang, [shujun@uow.edu.au](mailto:shujun@uow.edu.au), Fazhou Wang, [fzhwang@whut.edu.cn](mailto:fzhwang@whut.edu.cn)


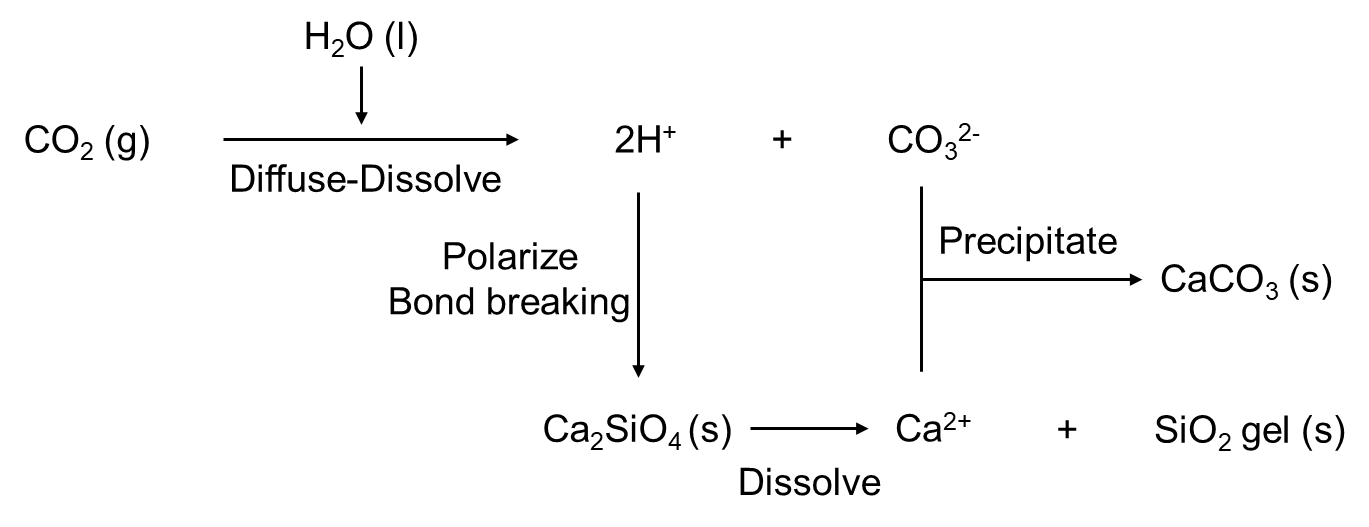
**Supplementary Figure 1│Chemical reaction process of in situ carbon mineralization in CMM.**

 **Supplementary Figure 2│Phase composition of CMM. (a)** X-Ray Diffraction (XRD) patterns and **(b)** phase assemblage of the biomimetic CMM.

**
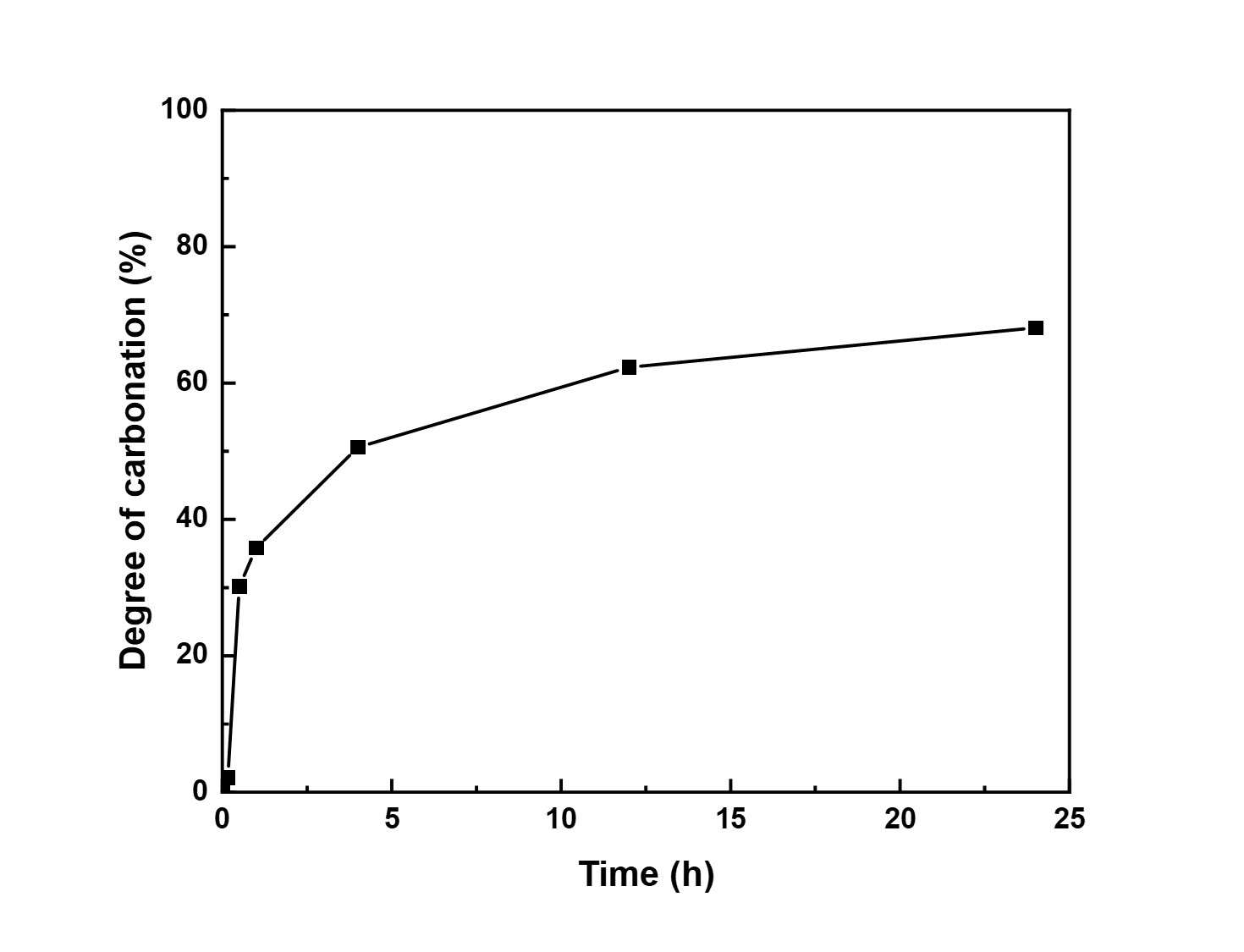
**

**Supplementary Figure 3│Degree of carbonation of CMM at different curing ages.**

**Supplementary Figure 4│The fatigue tests of the biomimetic CMM. (a)** Partial stress-time curves of cyclic loading tests. **(b)** Stress-strain curves of the specimen after 10,000 cyclic loading tests and the initial specimen.

**Supplementary Figure 5│Zeta potential of CaCO_3_ and gelatin.**

**
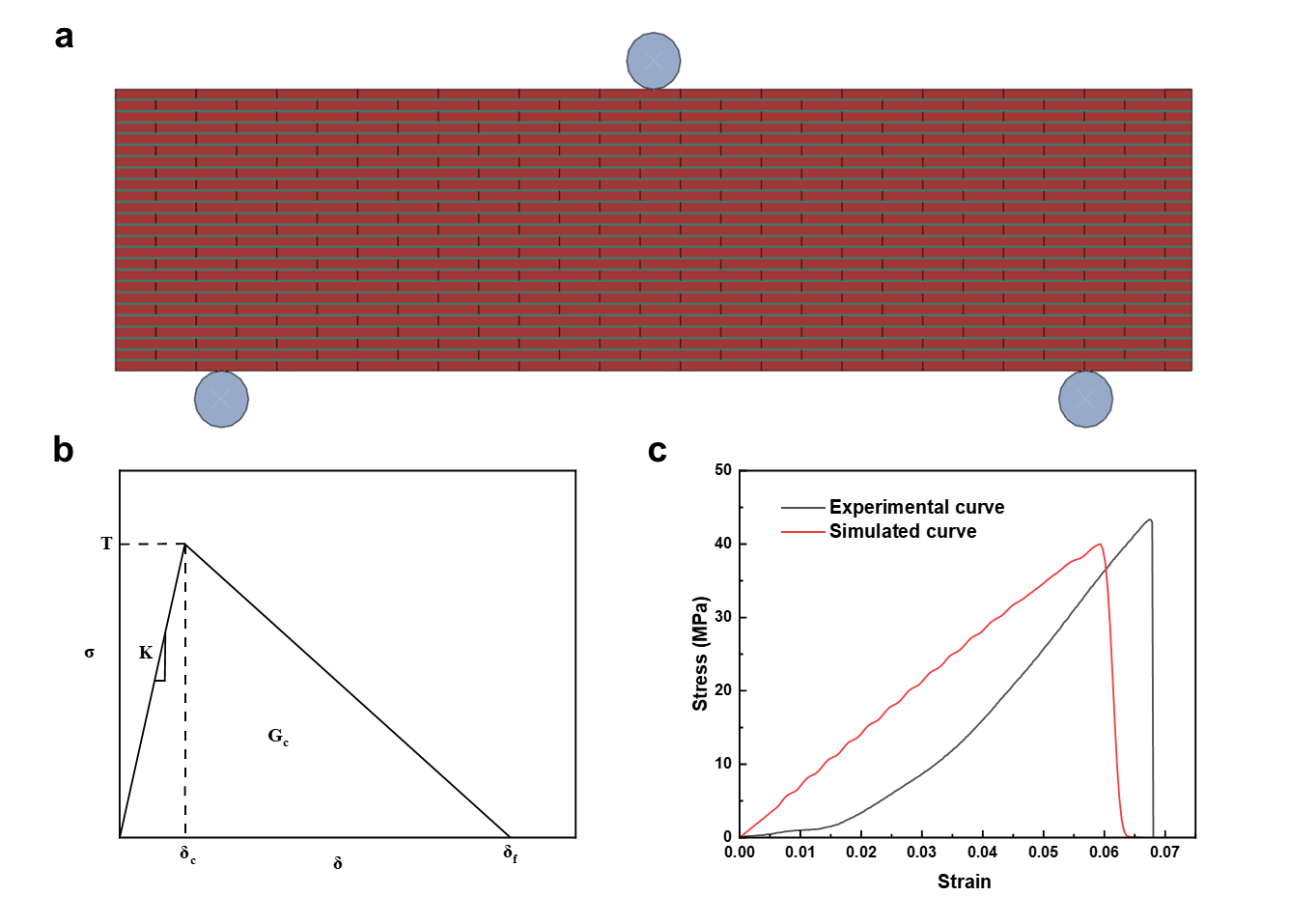
**

**Supplementary Figure 6│Schematic of the Abaqus simulation model. (a)** The initial configuration of the “brick-and-mortar” architecture in nacre-like hierarchical CMM under three-point bending. **(b)** Bilinear traction-separation response of the cohesive element. **(c)** The simulated vs. experimental stress-strain curves of the biomimetic CMM.

**Supplementary Figure 7│The high-speed video snapshots of fracture during mechanical testing.**
