# Supplementary material for: Energy‐Efficient Fabrication of Biomimetic Materials for Sustainable Infrastructure Applications
Source: Adv Sci (Weinh). 2025 Jun 25;12(35):e03854. doi: 10.1002/advs.202503854 (PMC12463093; doi:10.1002/advs.202503854)
Supplement: Supplementary file 2 — Supporting Information [file ADVS-12-e03854-s002.pptx]

## Slide 1
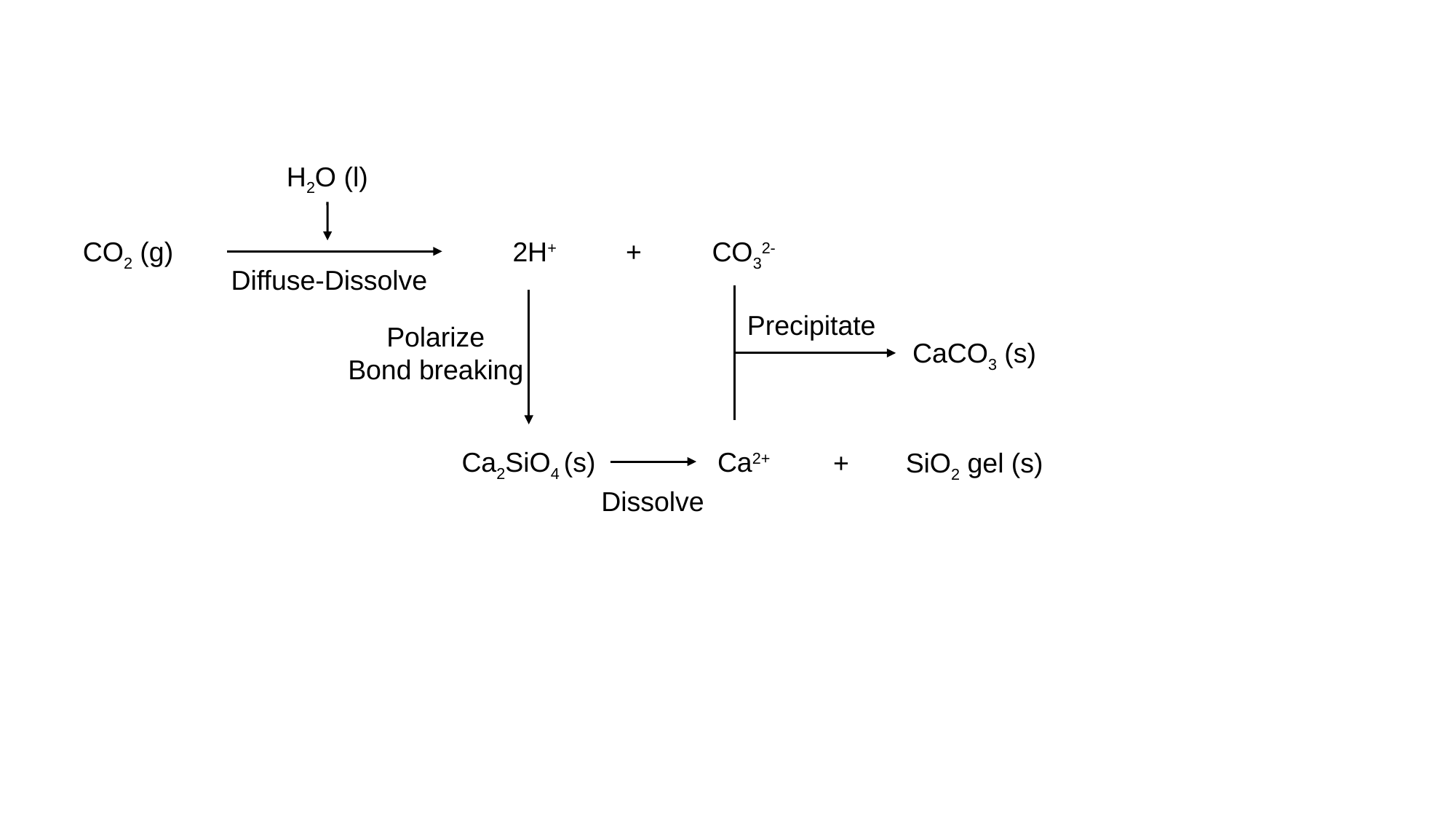

H2O (l)
CO2 (g)
2H+
+
CO32-
Diffuse-Dissolve
Precipitate
Polarize
Bond breaking
CaCO3 (s)
Ca2+
Ca2SiO4 (s)
+
SiO2 gel (s)
Dissolve

## Slide 2
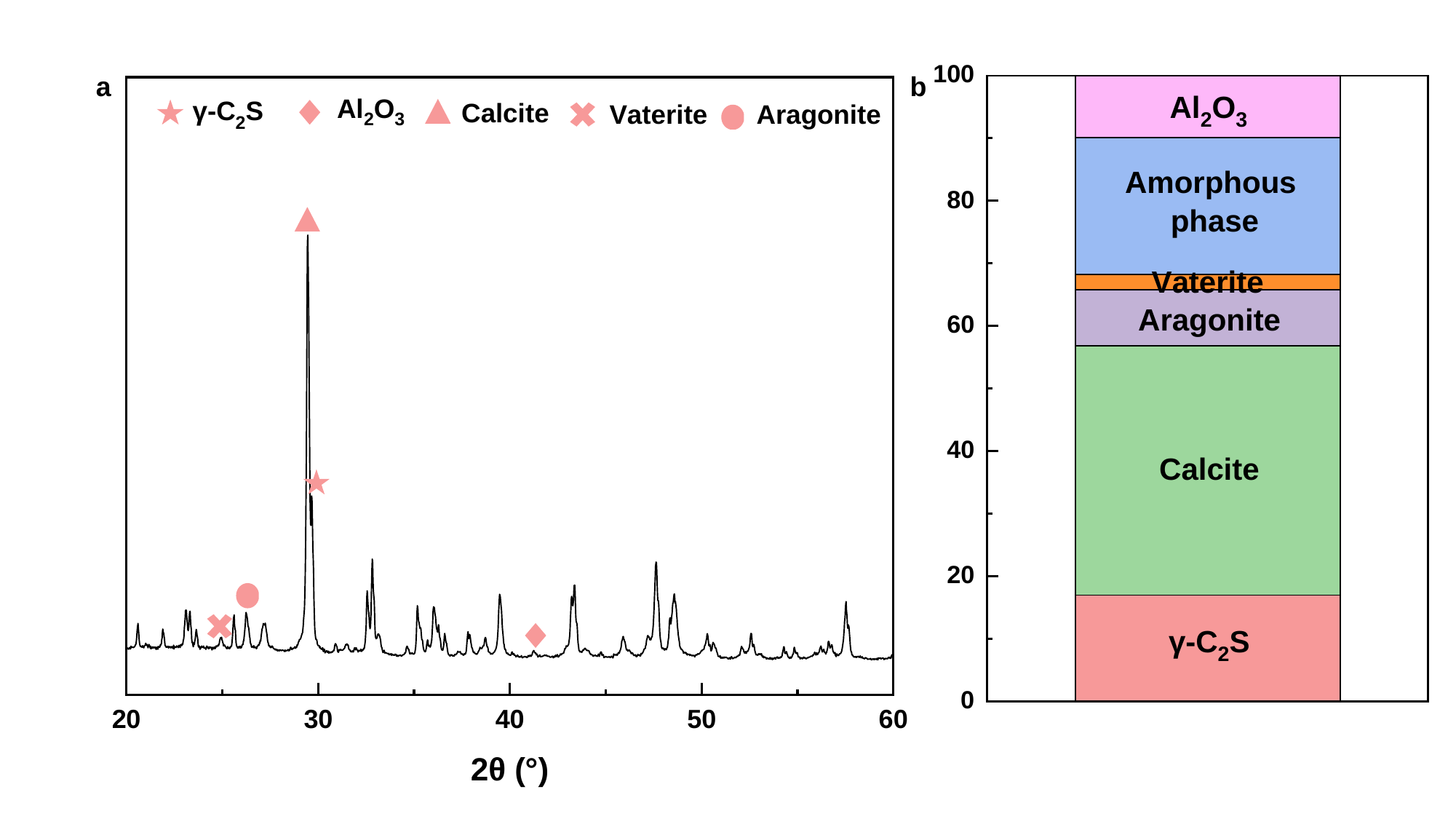

a
b

## Slide 3
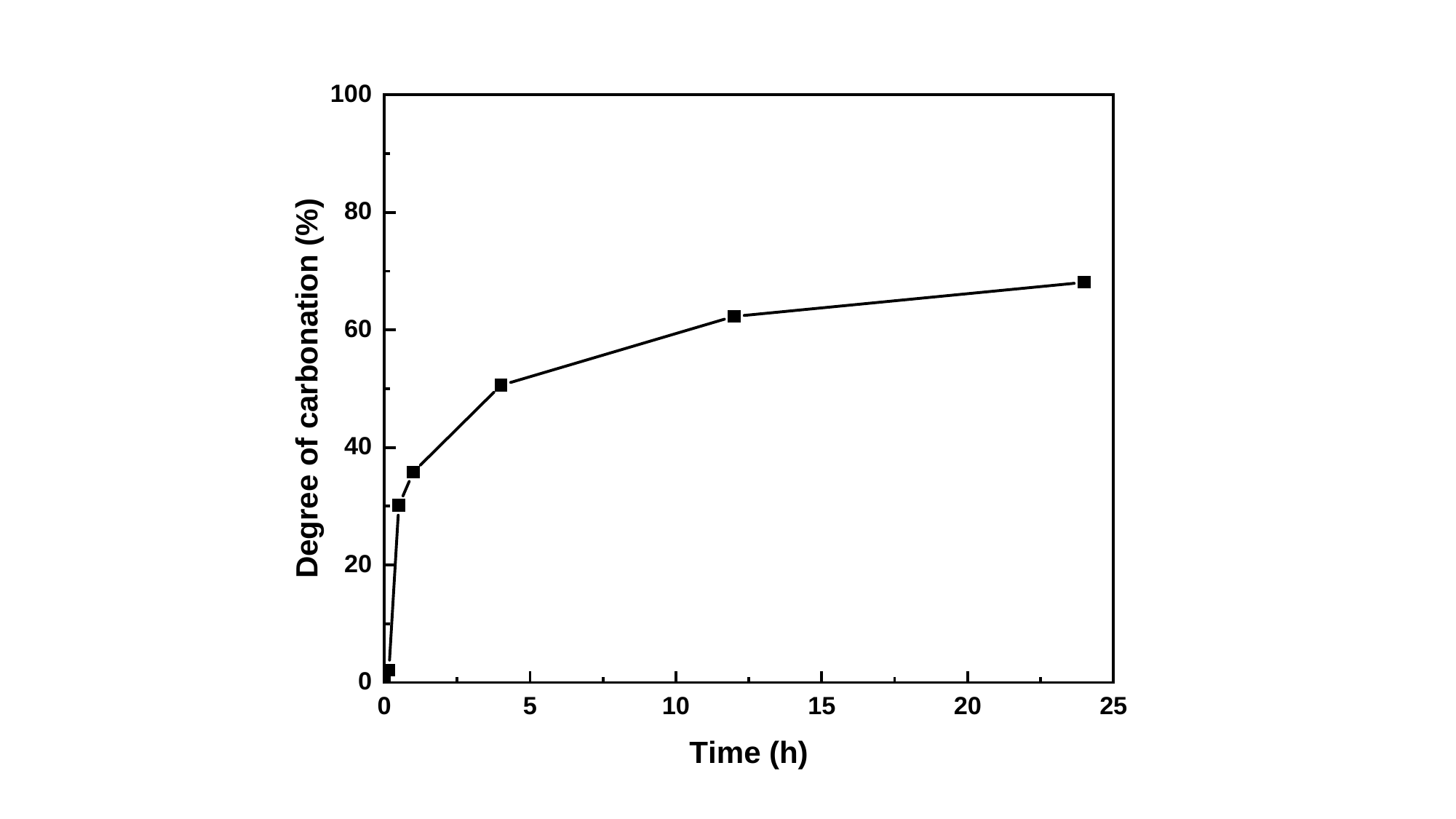

## Slide 4
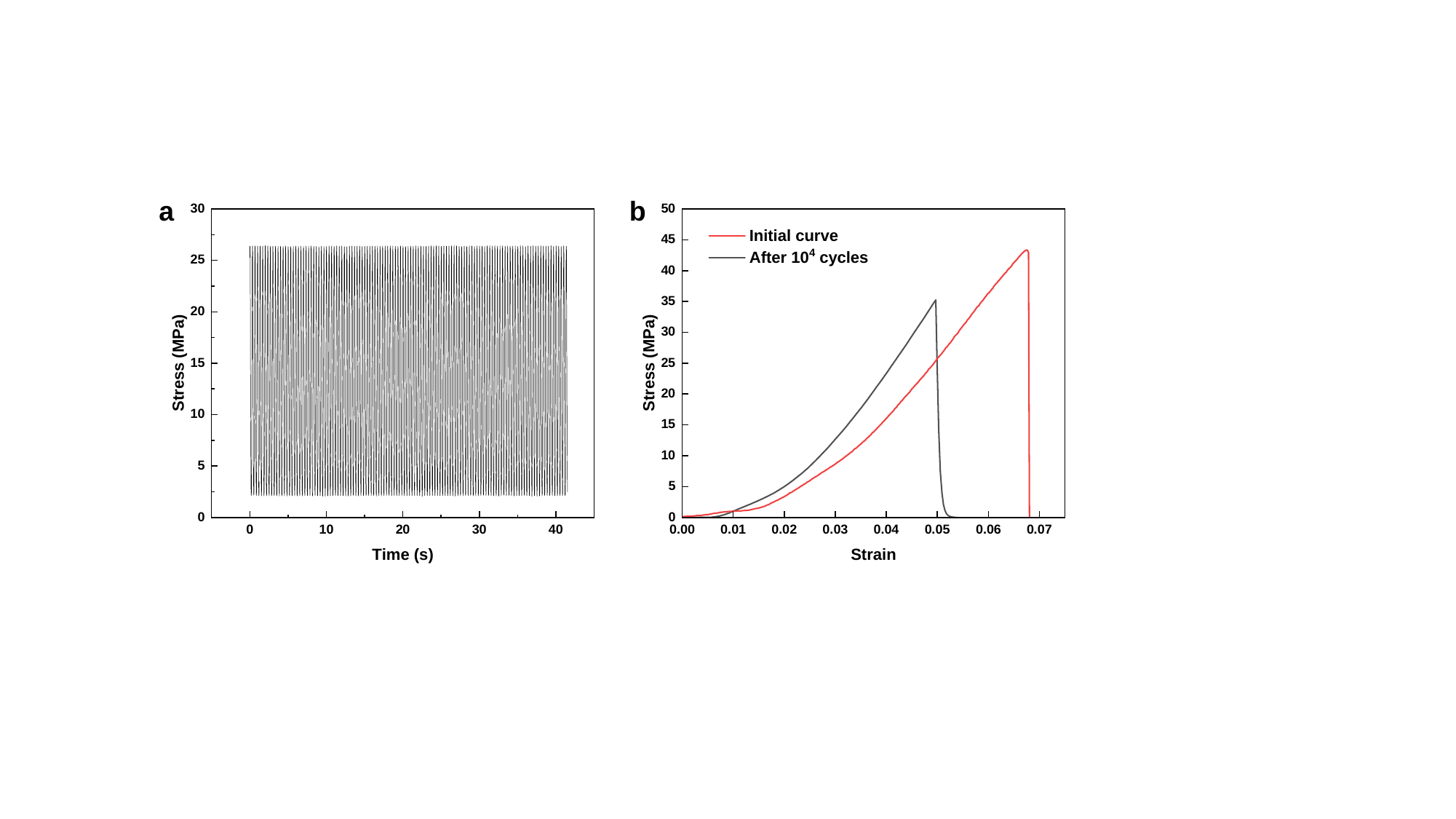

a
b

## Slide 5
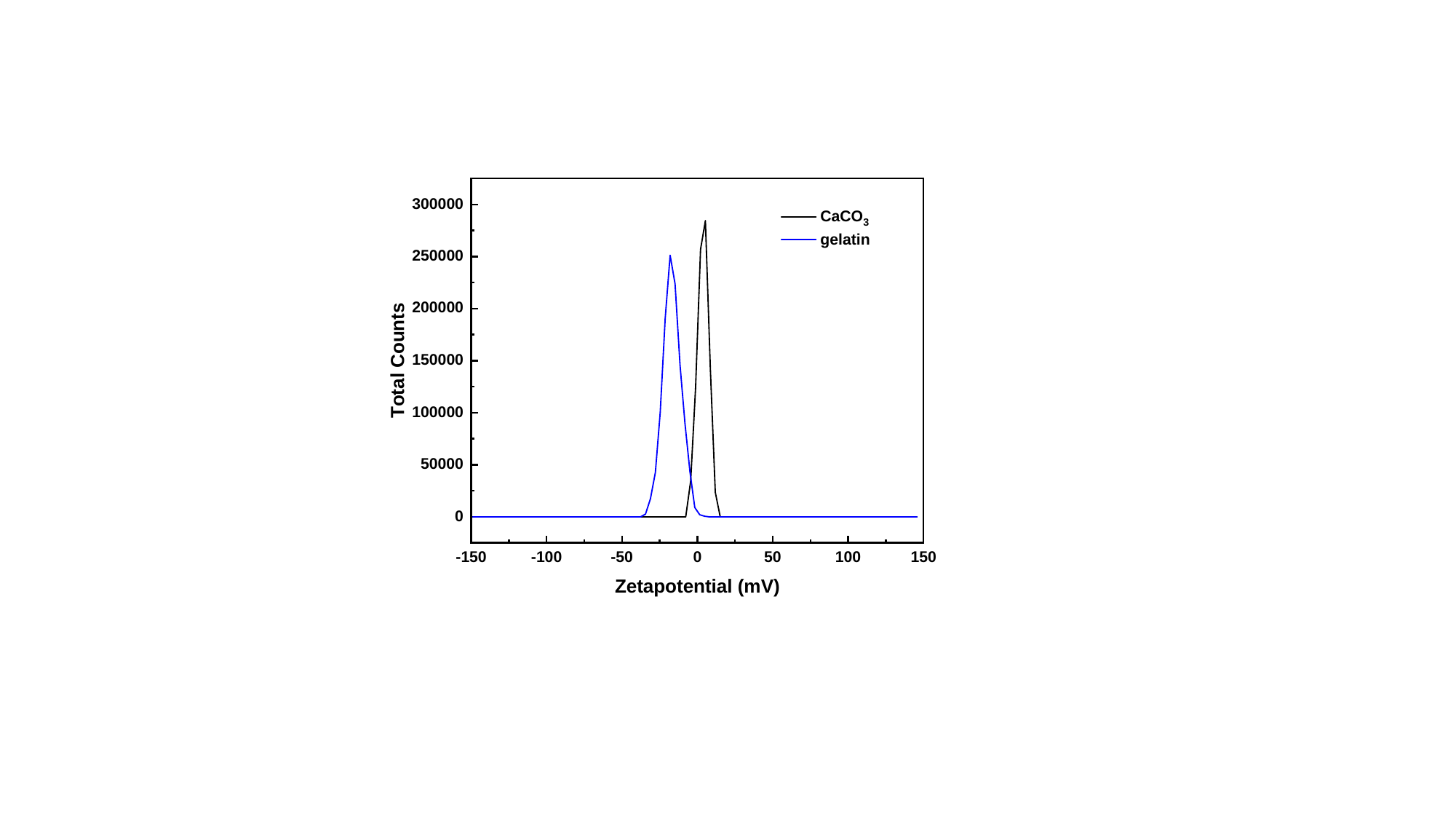

## Slide 6
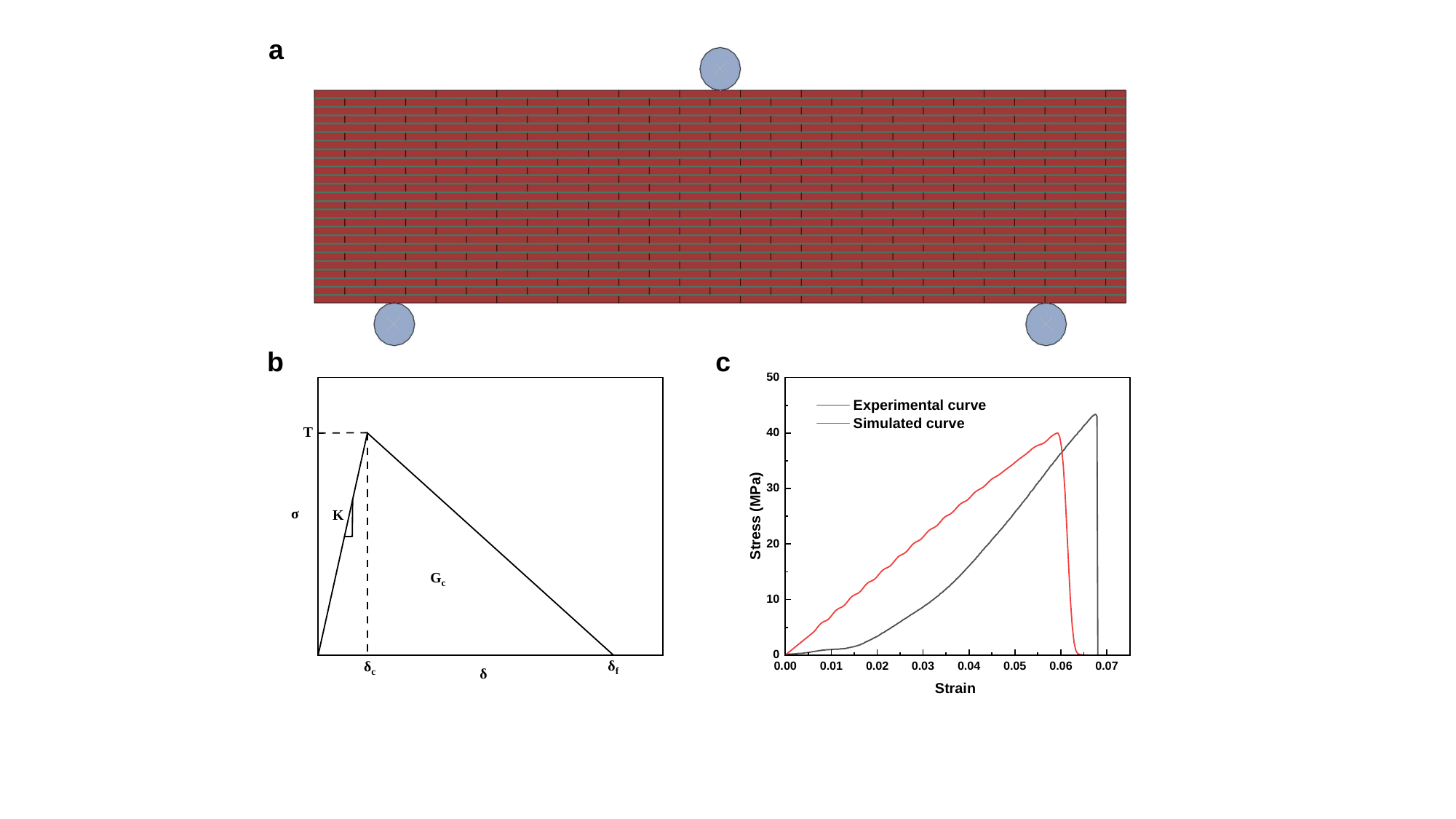

a
b
c

## Slide 7
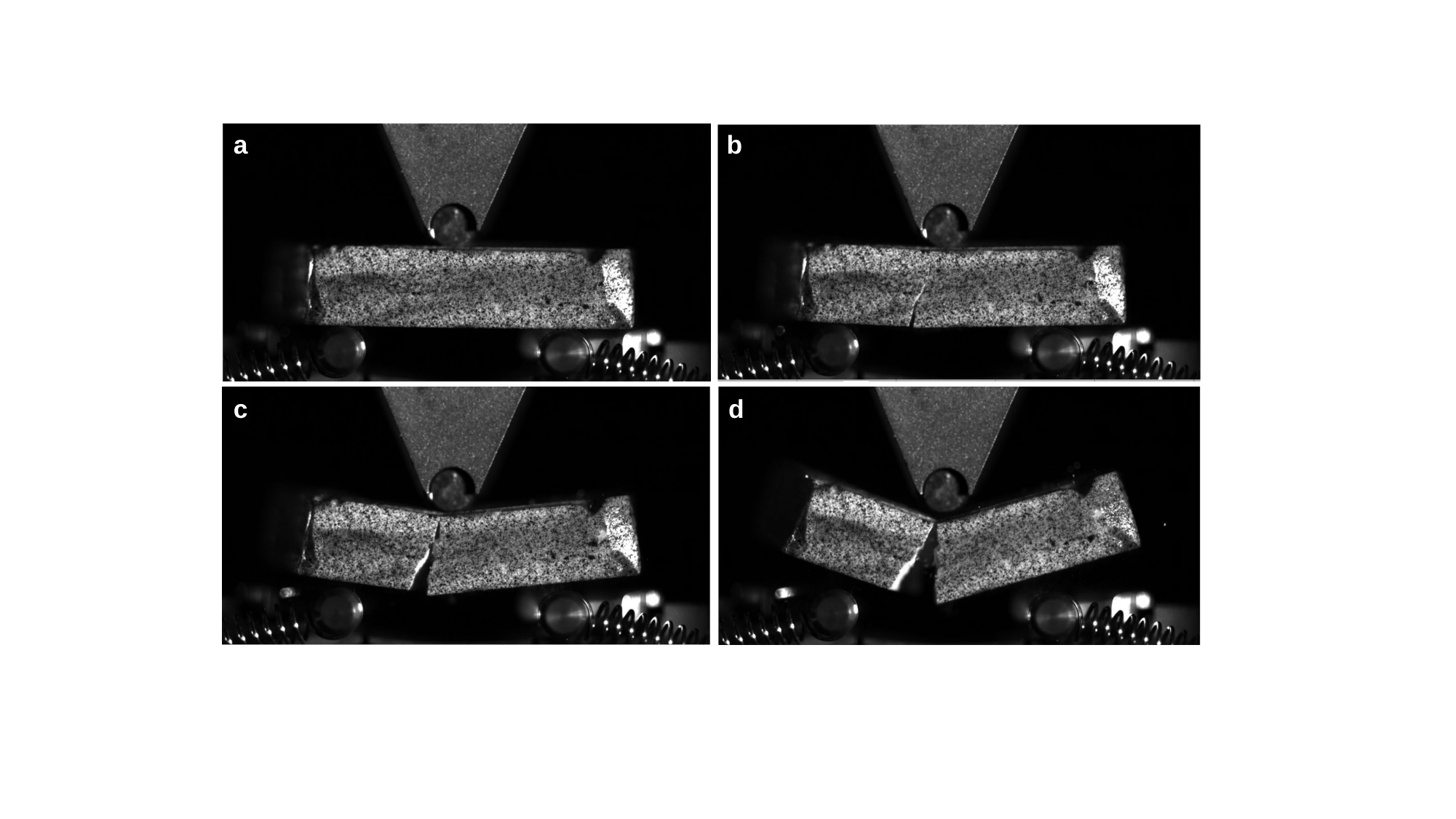

a
b
c
d
